# The Impact of Changes regarding Working Circumstances during COVID-19 Pandemic upon Patients Evaluated for Thyroid Dysfunction

**DOI:** 10.3390/ijerph19169856

**Published:** 2022-08-10

**Authors:** Anca Popa, Aurelia-Ioana Chereji, Monica Angelica Dodu, Ioan Chereji, Andreea Fitero, Cristian Marius Daina, Lucia Georgeta Daina, Dana Badau, Daniela Carmen Neculoiu, Carmen Domnariu

**Affiliations:** 1 Department of Endocrinology, Emergency Clinical County Hospital of Oradea, 410169 Oradea, Romania; 2 Department of Animal Science and Agritourism, Faculty of Environmental Protection, University of Oradea, 410087 Oradea, Romania; 3 Faculty of Medicine and Pharmacy, University of Oradea, 410087 Oradea, Romania; 4 Department of Infectious Diseases, Emergency Clinical County Hospital of Oradea, 410169 Oradea, Romania; 5 Petru Maior Faculty of Sciences and Letters, George Emil Palade University of Medicine, Pharmacy, Sciences and Technology, 540142 Targu Mures, Romania; 6 Interdisciplinary Doctoral School, Transilvania University, 500068 Brasov, Romania; 7 Faculty of Medicine, Transilvania University, 500068 Brasov, Romania; 8 Faculty of Medicine, Lucian Blaga University, 550169 Sibiu, Romania

**Keywords:** thyroid dysfunction symptoms, measures, lifestyle changes, symptoms, COVID-19 pandemic, work instability, endocrinology disorder

## Abstract

We evaluated patients who presented with thyroid dysfunction correlated symptoms that started when the Government took important measures to reduce the spread of COVID-19. These measures have influenced the safety of many people’s jobs. Data were collected from 378 patients that were clinically evaluated at the Endocrinology Department, between September 2020 and January 2021. Their health status modifications were statistically analyzed in correlation with their life and work changes. These changes were induced by measures associated with the COVID-19 pandemic. The lifestyle changes correlated with the COVID-19 pandemic have been present in both categories of patients: euthyroid and dysthyroid patients; 87.50% of euthyroid patients physically felt the pandemic-induced changes in their lives. It resulted in changes in lifestyle and job insecurity has a statistically significant influence (*p* < 0.01) on their state of health. The presence of life/work changes in men is strongly reflected in their state of health (*p* = 0.0004). Work instability that occurred as a side effect of the COVID-19 pandemic induced symptoms that made many people believe they have an endocrinology disorder.

## 1. Introduction

In the beginning, a few patients from Wuhan in China presented with pneumonia of unknown etiology. The situation was reported to the Chinese Center for Disease Control at the end of 2019. Initial investigations identified a novel coronavirus as the etiological agent [1,2,3]. The infection was correlated with the seafood and animal market in Wuhan and there was a suspicion that the transmission was linked to animals [4,5]. Rapidly, the infection was diagnosed in patients outside Wuhan city; those people did not have exposure to animal markets, suggesting person-to-person transmission [6,7,8].

Based on the agreement between WHO, the World Organization for Animal Health and the Food and Agriculture Organization of the United Nations (FAO), a name was established—COVID-19—that did not refer to a geographical location, an animal, an individual, or group of people, and which is also pronounceable and related to the disease [9]. Severe acute respiratory syndrome coronavirus 2 (SARS-CoV-2) marked its importance to global health in January 2020, when the World Health Organization (WHO) declared the coronavirus disease 2019 (COVID-19) outbreak a global health emergency and then, in March 2020, it became a global pandemic (1). The novel coronavirus was found to be highly contagious and rapidly spread across the world within 2–3 months [10,11]. COVID-19 started in Romania in February 2020 and had the same increasing trend observed worldwide [12,13].

The excessive spread of COVID-19 worldwide and on a national level led the Romanian authorities to take measures with great impact on social-economic activity: the closure of schools, restaurants, hotels, cafés, gradual closure of borders, limitation of people traveling from one area to another, no groups larger than three people on the streets, etc. It was not allowed to be outside the house from 10 P.M. to 6 A.M. All shopping centers were closed, except for food or pharmaceutical shops. With few exceptions, it was forbidden for foreign citizens and stateless persons to enter Romanian territory. Movement outside the home was prohibited, with some exceptions (work—if online activity was not possible, buying food or medicine, etc.). People over 65 were allowed to leave their homes only between 11 A.M. and 1 P.M. [14]. All these decisions instituted a national lockdown for 2 months. From May 2020, restrictions began to be a little more permissive, but strongly correlated with the epidemiological statistics regarding COVID-19. Until 1 March 2022, more than 2.7 million cases of COVID-19 were confirmed in Romania and over 61,000 deaths have been determined by SARS-CoV-2 infection [15].

Even if this strategy of social limitation is an important measure to reduce or limit the exponential rise of COVID-19 cases, it had an important impact on the economy, psyche and daily living habits of society [16,17,18].

The COVID-19 pandemic became an important psychological stress source. The risk of infection by COVID-19 increased social isolation and insecurities related to the present and future [19]. A major event, such as the COVID-19 pandemic may change people’s behaviors during the event in the immediate transition after the pandemic and years later [20]. The COVID-19 pandemic represented the main reason for staying at home and doing less in terms of social interactions, active work and economic benefits. This had a negative effect on people’s physical and mental health [21]. Even if the “great waves” of the pandemic have gone, the effects are still present and will broadly and substantially impact the economic, political and social situation (including the mental health of populations) worldwide. The course of the COVID-19 pandemic is yet to be determined and maybe new waves of infection will occur; it is important to understand that emotional and behavioral attitudes influence the course of pandemics [22].

The COVID-19 pandemic has and probably will continue to result in negative mental health outcomes, such as depression, anxiety and traumatic stress in people and populations throughout the world [23,24]. This is the reason why it is important to monitor the present and future effects of the COVID-19 pandemic on people‘s health. Even if there are many studies that reflect the impact of the COVID-19 pandemic on our life (from many points of view: health, social, economic, etc.), this research follows the indirect implications of the new rules associated with the pandemic on previously working people. Additionally, the study reflects the way that stress hides behind thyroid dysfunction-like symptoms. Our research did not identify any other study that correlates pandemic stress with thyroid dysfunction symptoms.

Therefore, the current study aimed to evaluate the impact of the COVID-19 pandemic-induced lockdown on patients presented for an endocrine examination, with an objective to assess their perception of their own state of health and to identify the etiology of their symptoms. We also searched for the correlation between symptomatology and job-induced modifications during the COVID-19 pandemic and how working insecurity can determine different kinds of symptomatology (thyroid-like symptoms).

## 2. Materials and Methods

### 2.1. Study Design

The present research has been conducted using an observational analytical study [25]. The study took place at the Emergency Clinical County Hospital of Oradea—The Endocrinology Department. It included 378 patients from the North West Region of Romania that were clinically evaluated for the first time in our department between September 2020 and January 2021. The cohort is represented by all the new cases that came for thyroid evaluation during the above-mentioned period of time. Given the high incidence of COVID-19 infection, the public reaction, and policy measures, the time frame of data collection is influenced by all these aspects. The data were collected at the end stage of the first year of the pandemic in Romania. The entire evolution of the pandemic correlated with the public information campaign regarding mortality and morbidity that described the disease and highlighted the importance of respecting social isolation, which induced a state of fear and reduced the number of patients presenting for medical evaluation for non-respiratory diseases [26,27,28].

Patients were clinically and para-clinically examined based on their symptoms correlating with the thyroid (including laboratory analysis and thyroid US). All the results were archived in the patients’ files and these files were reanalyzed for this study. According to the outcomes of their investigations, the group was divided into two subgroups: euthyroid patients and patients with thyroid dysfunction. Several factors that could have induced their symptoms were analyzed (including working habits). With regards to reducing the infection risk, there were work changes that decreased contact with other people, which included remote work, reduced working hours, or temporary and permanent layoffs and business closures [26]. All patients went through the changes induced by the COVID-19 pandemic.

### 2.2. Participants

From the total of 378 patients, 224 patients were women (59.26%) and 154 patients were men (40.74%). The mean age of all patients was 46.8 years. Demographic characteristics of the cohort are presented in Table 1. Based on the outcomes of their investigations, they were divided into two groups: euthyroid patients (296 patients) and patients with thyroid dysfunction (82 patients).

Study Exclusion criteria: children or patients younger than 18 years of age, patients older than 65 years of age, menopausal or premenopausal women, patients with known endocrine disorders in their personal medical history, and patients with acute respiratory infection at the moment of evaluation.

### 2.3. Measures

Every patient went through an examination. All patients included in the study were examined by US of the thyroid. Thyroiditis refers to chronic autoimmune thyroiditis (Hashimoto’s Thyroiditis) when TPO Ab levels are elevated above the normal range. During their examination, patients were asked if they had a COVID-19 infection. Each patient gave his/hers consent for examination and evaluation. The following clinical and paraclinical aspects were registered for all study participants:

— symptomatology,
— family history of endocrine diseases,
— life and work style,
— personal medical history,
— treatment,
— clinical general examination (including weight and height),
— blood tests (complete blood count, TSH, FT4, Thyroid peroxidase antibodies—TPO, serum Calcium),
— thyroid ultrasound,
— patients’ status of health was analyzed in comparison to their gender and their employment situation.

Values of hormones were determined using Chemiluminescent microparticle immunoassay (CMIA). Normal ranges: TSH: 0.27–4.2 µUI/mL; FT4: 0.89–1.78 ng/dl; TPO Ab < 34 UI/mL. Subclinical hypothyroidism is defined as a state of increased serum thyroid-stimulating hormone (TSH) levels, with circulating free thyroxine (FT4) concentrations within the reference range. Overt thyroid dysfunction means that both the TSH levels and the thyroid hormone levels (FT4) are abnormal. These definitions are in accordance with the European Thyroid Association Guidelines and the American Thyroid Association Guidelines.

### 2.4. Statistical Analysis

The IBM Statistical Package for Social Sciences (SPSS) Statistics for Windows, was used for analysis. The data collected during the study were statistically analyzed using binary logistic regression models to estimate the impact of public health measures on patients. Full information on all variables of interest was available for all the patients that we have had evaluated. Therefore, there was no use for imputation methods and only analyzed complete cases. These cases form the basis of our analysis of the state of health of the patients presented at the Endocrinology Department.

Measurement data with a normal distribution were expressed as mean ± standard deviation (x ± s); also, data were presented as percentage (%). The distribution of prevalence rates and mean scores of the clinical measures were studied using the chi-square test and one-way analysis of variance (one-way ANOVA). Comparison between groups was performed using t-tests, and *p* was considered to indicate statistically significant differences if the value of *p* was <0.05.

## 3. Results

When asked about the reason for the endocrinological checking and their symptomatology, patients complained especially about feeling a “lump in the throat” or sensation of “constriction” in the neck, dyspnea, anxiety, palpitations, weight changes, etc. (Table 2). The symptoms presented in Table 2 are symptoms accused by the patients that were sent for an endocrinological evaluation. Many of those symptoms are parts of the clinical presentation of thyroid disease. All these symptoms do not represent the typical portrait of thyroid disease, but it is rarely the case in practice to meet a ”typical patient”.

In all patients, the symptoms started 3–6 months before their endocrinological evaluation (representing the time interval between March and August 2020); 18.3% of patients confirmed that they had at least one first-degree relative suffering from thyroid disease. When patients were asked about their life and work conditions during the last half year, 87.30% of them mentioned changes in their lifestyle, especially correlated with their jobs (Table 3).

After clinical evaluation, patients were asked to do blood tests and a thyroid ultrasound. The paraclinical evaluations determined thyroid disorders in 82 patients (21.69%): 24 men and 58 women (Table 4). The results were integrated into four categories correlated with four thyroid pathologies (that can be considered as etiology for the above-mentioned symptoms [29]: nodular goiter, hypothyroidism, hyperthyroidism, and thyroiditis (patients with two disorders, have been considered only once for the statistical calculus). Patients with no thyroid dysfunction were also investigated for ENT or infectious disease to eliminate any organic causes for their symptoms.

Table 5 shows that the lifestyle changes induced by the COVID-19 pandemic have been present in both categories of patients: euthyroid and dysthyroid patients; 87.50% of euthyroid patients physically felt the pandemic-induced changes in their lives. In terms of lifestyle changes impacting the state of health, the results observed (Table 5) are considered to be statistically significant (*p* < 0.01).

The presence of life/work changes in men is strongly reflected in their state of health (*p*-value equals 0.000—this difference was considered to be extremely statistically significant). In women, endocrinological checking was correlated with symptoms determined by events in their life during pandemics, but not as strongly influenced as in men (difference was considered to be statistically significant, *p* = 0.032) (Table 5).

## 4. Discussions

Previous studies that investigated the psychological impact of the COVID-19 pandemic have focused mainly on stress, anxiety, and depression (using surveys) to measure their prevalence, but not on the fact that stress can be somatized and may be expressed as an endocrine symptom. This study is one of the first to investigate the correlation between working instability and thyroid pathology as a clinical consequence of COVID-19. The aspects and results analyzed in this study with regard to working people and their gender, may be useful in determining not only appropriate medical services but also to develop instruments to help those that may be collateral victims of a pandemic [30].

After the rapid spread of COVID-19 in the world, and in Romania as well, several measures to control and prevent the disease were taken by health and governmental authorities. The most widespread measure by the authorities was social distancing [31], generally understood by the population and the media as social isolation [32]. Workplaces were considered to be one of the high-risk areas for COVID-19 transmission. Therefore, home office work was encouraged if possible [33,34]. In these conditions, work habits and especially the security of a stable job have changed during the COVID-19 pandemic.

According to the International Labor Organization [35] globally, the decline in working hours in 2020 translated into both employment losses and a reduction in the working hours for those who remained employed. By the first trimester of 2020, over 10 million people from the United States had filed for unemployment due to the pandemic’s restrictions: closing industries having ripple effects across the world [36]. Globally, there were 114 million job losses in 2020 relative to 2019. Unemployment was higher for women (5.0%) than for men and for young workers (8.7%) than for older workers.

All patients that were included in the study presented different kinds of symptoms, especially correlating with neck discomfort (lump in the throat, constriction, difficulty of breathing). This was an important reason for being referred to an endocrinologist by their family doctor. Another argument for an endocrinological exam is the possibility that patients who suffered from SARS-CoV-2 may have reduced thyroid function [20,37,38], with potential thyroid cellular access by the virus. Both follicular and parafollicular cells of the thyroid gland can be extensively destroyed by the virus [39,40].

Other relevant symptoms were fatigue, weight changes, insomnia, palpitations, and headache—these symptoms were described by more than 50% of the patients (independent of gender). Clinical and paraclinical investigation showed that only 21.69% of these patients had an endocrine (thyroid) disease that could explain and determine their symptoms. For the rest of them (78.31%—296 patients) no physical or functional problem was identified. The statistical study reveals the high probability (*p* = 0.0091) that the symptoms are due to the life changes experienced by the patients during the COVID-19 pandemic. Physical symptoms of stress may include headache, a sensation of fatigue, leg pain, tachycardia, and at the same time pain that can result from emotional stress [41]. The economic fallout of the COVID-19 pandemic resulted in unprecedented job losses, which impaired mental well-being significantly [42].

At the same time, even if these patients have symptoms induced by stress, it is important to acknowledge that psychological stress is a risk factor for many common diseases, such as somnipathy, depression, coronary heart disease, obesity, and type-2 diabetes [19]. It is a fact that women seek medical consultations more frequently than men [43]. Additionally, thyroid diseases affect women 500% more than males [44]. In our study, the reported ratio of women vs. men is 1.45:1. Out of 224 women, 58 of them (25.89%) are confirmed with a thyroid problem. In the male group, there are 21 men (13.63%) with a thyroid disease. At the same time, 148 men (96.10%) confirm changes in their jobs, and only 81.25% of women complain of the same problem. The insecurity regarding their workplace has a greater impact on men than on women, and this insecurity is manifested through physical symptoms. Meanwhile, a Turkish cross-sectional study, suggests that women are more likely to be psychologically affected by the COVID-19 pandemic, alongside individuals with previous psychiatric illness, individuals living in urban areas and those with an accompanying chronic disease [45].

The variation in mental distress according to gender and ethnicity is supported by a study conducted in the UK, which shows that women and Black, Asian, and minority ethnic men experienced a higher increase in mental distress than White British men [46].

Our study reveals the impact of modifying working conditions or even losing a job due to the COVID-19 pandemic on people. The results of the present study are consistent with other studies developed around the world. There are many studies showing that job/work-induced modifications by the COVID-19 pandemic have negatively affected the population. In Germany and Switzerland, 31% percent of participants in a survey strongly agreed that their work life had worsened and 30% strongly agreed that their private life too had worsened [47]. This negative impact brings a wide range of mental health problems including anxiety, depression and stress-related symptoms or disorders, and psychiatric morbidity (paranoid ideation, hallucinations, and insomnia that may occur in the general public) [48,49].

Most of these studies reflect people’s health state using surveys [30,50,51,52] to understand the magnitude of the problem, while we identified that stress induced by the COVID-19 pandemic is somatized as thyroid correlated symptoms. The analysis of the complexity of the effects of the COVID-19 pandemic on health, lifestyle, work conditions and psychological and social aspects must be addressed interdisciplinarily and transdisciplinarily [53,54,55,56,57,58,59,60].

### 4.1. Strengths

The present study analyzes, in an integrative way, the complexity of the effects induced by the COVID-19 pandemic aiming at a series of relevant parameters, such as thyroid dysfunction correlated symptoms related to the measures to reduce the spread of COVID-19, a large number of investigated subjects, family history of endocrine diseases, life and work style, personal medical history, treatment, clinical general examination (including weight and height), blood tests (complete blood count, TSH, FT4, Thyroid peroxidase antibodies—TPO, serum Calcium), thyroid ultrasound.

### 4.2. Limitations

In the study, we did not analyze all components of lifestyle, work-related changes, and other health parameters of patients with thyroid symptoms who experienced changes induced by the COVID-19 pandemic.

## 5. Conclusions

The COVID-19 pandemic represented not only a health problem but also induced a social-economic disorder. One of the most important measures adopted to limit the spread of COVID-19 was social isolation. Social isolation required modifications in lifestyle, but also in working habits. Many people started working from home (online) if it was possible, for some individuals work time was reduced, and there were people that lost their jobs or their work contract was temporally suspended.

This study’s results demonstrate the negative impact of the COVID-19 pandemic, which aligns with findings from other studies on past pandemics. Insecurity is correlated with an unstable workplace/job and determined different kinds of symptoms in people, such as a lump in the throat, constriction, difficulty of breathing, fatigue, weight changes, insomnia, palpitations and headache. Men were more affected than women. It is important for these patients to be evaluated by an endocrinologist, but most of them do not have any physical or functional abnormalities. If this situation persists, there is a possibility to develop serious health problems: heart diseases, diabetes mellitus, obesity, depression, etc.

Many people do not correlate their health state with the changes that occurred in their lives and search for help and answers in different kinds of pathologies (including endocrine diseases). A closer look at their medical history, life and work conditions can help reveal the real cause of their symptoms. The COVID-19 pandemic took many lives and made many victims due to SARS-CoV-2, but it also affected and made collateral victims through people that were not infected with the virus and supported and respected the rules induced by the pandemic.

## Figures and Tables

**Table 1:** Demographic characteristics of the patients.

Variables		Total		Men		Women	
		No.	%	No.	%	No.	%
Number		378	100	154	40.74	224	59.26
Mean age (years)		46.8 ± 6.5	-	50.2 ± 5.7	-	44.5 ± 6.6	-
Provenance	Urban	281	74.34	130	84.42	151	67.41
	Rural	97	25.66	24	15.58	73	32.59
Educational level	8 classes or less	22	5.83	8	5.19	14	6.25
	High school	186	49.21	79	51.30	107	47.77
	Bachelor	135	35.71	60	38.96	75	33.48
	Master or more	35	9.25	7	4.55	28	12.50
Employment	Yes	361	95.50	152	98.70	209	93.30
	No	17	4.50	2	1.30	15	6.70

**Table 2:** Patients’ symptomatology.

Patients	Total		Men		Women		Differences Man-Women	
Symptoms	No.	%	No.	%	No.	%	No	%
Lump/constriction sensation	378	100	154	40.74	224	59.26	−70	−18.52
Dyspnea	343	90.74	142	92.20	201	89.73	−52	2.47
Anxiety	217	57.41	39	25.32	178	79.46	−139	−54.14
Weight gain (more than 5 kg in 6 months)	224	59.26	66	42.86	158	70.54	−92	−27.68
Weight loss (more than 5 kg in 6 months)	40	10.58	19	12.34	21	9.38	−3	2.96
Insomnia	81	21.43	45	29.22	36	16.07	9	13.15
Perspiration	65	17.20	11	7.14	54	24.11	−43	−16.97
Fatigue	245	64.81	79	51.30	166	74.11	−87	−22.81
Palpitations	262	69.31	93	60.39	169	75.45	−76	−15.06
Headaches (frontal)	237	63.70	102	66.23	135	60.27	−33	5.96

**Table 3:** Changes in patients’ life/work conditions.

Patients	Total		Men		Women		Differences Men-Women	
Life/Work Conditions	No.	%	No.	%	No.	%	No	%
Suspension of professional activity	104	27.51	57	37.01	47	20.98	10	16.03
Reduction of professional activity	69	18.25	25	16.23	44	19.64	−19	−3.41
Online activity	112	29.63	39	25.32	73	32.59	−34	−7.27
Mixt activity	29	7.67	14	9.09	15	6.70	−1	2.39
Job change	16	4.23	13	8.44	3	1.34	10	7.10
No (significant) life/work changes	48	12.70	6	3.90	42	18.75	36	14.85

**Table 4:** Thyroid dysfunctions identified in patients.

Patients	Total		Men		Women		Differences Men-Women	
Thyroid Pathology	No.	%	No.	%	No.	%	No	%
Nodular goiter	37	9.79	15	9.74	22	9.82	−7	−0.08
Hypothyroidism	20	5.29	1	0.65	19	8.48	−18	−7.83
Hyperthyroidism	3	0.79	1	0.65	2	0.89	−1	−0.24
Thyroiditis	22	5.82	7	4.54	15	0.70	−8	3.84

**Table 5:** Comparison between patients with euthyroidism and those with dysthyroidism regarding their lifestyle changes.

Parameters	Total		Men		Women	
	Euthyroid	Dysthyroid	Euthyroid	Dysthyroid	Euthyroid	Dysthyroid
Total	296	82	130	24	166	58
Lifestyle changes	259	71	127	21	132	50
Mean	277.50	76.50	128.50	22.50	149.00	54.00
SD	26.16	7.78	2.12	2.12	24.04	5.66
SEM	18.50	5.50	1.50	1.50	17.00	4.00
*n*	2	2	2	2	2	2
*p*	0.009		0.000		0.032	
95% C.I. (lower; upper)	117.96; 284.04		96.87; 115.13		19.86; 170.14	
t	10.414		49.968		5.439	
df	2		2		2	
SE of difference	19.300		2.121		17.464	

t—Student test value, *p*—level of probability, SD—standard deviation, SEM—standard mean errors, CI—confidence interval, df—degree of freedom, SE—standard errors.

## References

[1] Rodriguez-Guerra M., Jadhav P., Vittorio T.J. (2021). Current treatment in COVID-19 disease: A rapid review. Drugs Context.

[2] Hasöksüz M., Kiliç S., Saraç F. (2020). Coronaviruses and SARS-COV-2. Turk. J. Med. Sci..

[3] Mohan B.S., Nambiar V. (2020). COVID-19: An Insight into SARS-CoV-2 Pandemic Originated at Wuhan City in Hubei Province of China. J. Epidemiol. Infect. Dis..

[4] Gavriatopoulou M., Ntanasis-Stathopoulos I., Korompoki E., Fotiou D., Migkou M., Tzanninis I.G., Psaltopoulou T., Kastritis E., Terpos E., Dimopoulos M.A. (2021). Emerging treatment strategies for COVID-19 infection. Clin. Exp. Med..

[5] Habibzadeh P., Stoneman E.K. (2020). The novel coronavirus: A bird’s eye view. J. Occup. Environ. Med..

[6] Ashour H.M., Elkhatib W.F., Rahman M.M., Elshabrawy H.A. (2020). Insights into the recent 2019 novel coronavirus (SARS-CoV-2) in light of past human coronavirus outbreaks. Pathogens.

[7] Xu J., Zhao S., Teng T., Abdalla A.E., Zhu W., Xie L., Wang Y., Guo X. (2020). Systematic Comparison of Two Animal-to-Human Transmitted Human Coronaviruses: SARS-CoV-2 and SARS-CoV. Viruses.

[8] Cui J., Li F., Shi Z.L. (2019). Origin and evolution of pathogenic coronaviruses. Nat. Rev. Microbiol..

[9] WHO (2020). Director-General’s Remarks at the Media Briefing on 2019-nCoV. https://www.who.int/director-general/speeches/detail/who-director-general-s-remarks-at-the-media-briefing-on-2019-ncov-on-11-february-2020.

[10] Muralidar S., Ambi S.V., Sekaran S., Krishnan U.M. (2020). The emergence of COVID-19 as a global pandemic: Understanding the epidemiology, immune response and potential therapeutic targets of SARS-CoV-2. Biochimie.

[11] Atzrodt C.L., Maknojia I., McCarthy R.D.P., Oldfield T.M., Po J., Ta K.T.L., Stepp H.E., Clements T.P. (2020). A Guide to COVID-19: A global pandemic caused by the novel coronavirus SARS-CoV-2. FEBS J..

[12] Romanian Ministry of Health Newsletter (2020). https://fra.europa.eu/sites/default/files/fra_uploads/romania-report-covid-19-april-2020_en.pdf.

[13] (2022). Official Data, COVID-19 Actual Data. https://datelazi.ro/.

[14] Romanian Ministry of Internal Affair, Military Ordinance No. 3/24.03.2020 on Measures to Prevent the Spread of COVID-19. https://www.mai.gov.ro/wp-content/uploads/2020/03/MILITARY-ORDINANCE-on-measures-to-prevent-the-spread-of-COVID-19.pdf.

[15] Romanian Ministry of Internal Affair, COVID-19 Information, Strategic Communication Group, 1st of March 2022. https://www.mai.gov.ro/informare-covid-19-grupul-de-comunicare-strategica-1-martie-ora-13-00-2/.

[16] (2020). Word Vision Romania, The Welfare of the Child in Rural Areas during Pandemic. https://worldvision.ro/2020/08/04/studiu-world-vision-romania-viata-copiilor-din-rural-in-pandemie-40-dintre-parinti-nu-au-reusit-sa-asigure-alimentele-si-produsele-de-baza-doar-60-dintre-elevi-au-facut-scoala-online.

[17] Grover S., Sahoo S., Mehra A., Avasthi A., Tripathi A., Subramanyan A., Pattojoshi A., Rao G.P., Saha G., Mishra K.K. (2020). Psychological impact of COVID-19 lockdown: An online survey from India. Indian J. Psychiatry.

[18] Varalakshmi R., Swetha R. (2020). Covid-19 lock down: People psychology due to law enforcement. Asian J. Psychiatry.

[19] Lei L., Tang J., Su D., Deng D., Huang X. (2021). Relevant Factors and Intervention Measures of Psychological Stress-Induced Hyperthermia among Medical Staff in Temporary COVID-19 Negative Pressure Wards. Iran. J. Public Health.

[20] Brancatella A., Ricci D., Cappellani D., Viola N., Sgrò D., Santini F., Latrofa F. (2020). Is Subacute Thyroiditis an Underestimated Manifestation of SARS-CoV-2 Infection? Insights from a Case Series. J. Clin. Endocrinol. Metab..

[21] (2020). WHO Campaigns, Healthy at Home. https://www.who.int/campaigns/connecting-the-world-to-combat-coronavirus/healthyathome?gclid=CjwKCAjwj42UBhAAEiwACIhADrPwJAlqcKzmyDFKcZxpUBln5mssmixmHdgSnJhMkihWBsBSY1NIyRoCYS8QAvD_BwE.

[22] Funk S., Salathe M., Jansen V.A. (2010). Modelling the influence of human behaviour on the spread of infectious diseases: A review. J. R. Soc. Interface.

[23] Boden M., Zimmerman L., Azevedo K.J., Ruzek J.I., Gala S., Abdel Magid H.S., Cohen N., Walser R., Mahtani N.D., Hoggatt K.J. (2021). Addressing the mental health impact of COVID-19 through population health. Clin. Psychol. Rev..

[24] Pandey K., Thurman M., Johnson S.D., Acharya A., Johnston M., Klug E.A., Olwenyi O.A., Rajaiah R., Byrareddy S.N. (2021). Mental Health Issues During and After COVID-19 Vaccine Era. Brain Res. Bull..

[25] Centre for Evidence-Based Medicine (2016). Study Designs. https://www.cebm.net/2014/04/study-designs/.

[26] Kerstin H., Wenz S.E. (2021). Education, health behavior, and working conditions during the pandemic: Evidence from a German sample. Eur. Soc..

[27] McGregor S., Henderson K.J., Kaldor J.M. (2014). How are health research priorities set in low- and middle-income countries? A systematic review of published reports. PLoS ONE.

[28] Danhieux K., Buffel V., Pairon A., Benkheil A., Remmen R., Wouters E., van Olmen J. (2020). The impact of COVID-19 on chronic care according to providers: A qualitative study among primary care practices in Belgium. BMC Fam. Pract..

[29] Walsh J.P. (2016). Managing thyroid disease in general practice. Med. J. Aust..

[30] Chima C., Shalaby R., Lawal M.A., Vuong W., Hrabok M., Gusnowski A., Surood S., Greenshaw A.J., Wells K., Agyapong V.I.O. (2022). COVID-19 Pandemic: Influence of Gender Identity on Stress, Anxiety, and Depression Levels in Canada. Trauma Cent..

[31] WHO (2022). Advice for the Public: Coronavirus Disease (COVID-19). https://www.who.int/emergencies/diseases/novel-coronavirus-2019/advice-for-public.

[32] Bezerra A.C.V., Silva C.E.M.D., Soares F.R.G., Silva J.A.M.D. (2020). Factors associated with people’s behavior in social isolation during the COVID-19 pandemic. Cien Saude Colet.

[33] Güner R., Hasanoğlu I., Aktaş F. (2020). COVID-19: Prevention and control measures in community. Turk. J. Med. Sci..

[34] Parker K., Horowitz J.M., Minkin R. (2021). How the Coronavirus Outbreak Has-and Hasn’t-Changed the Way Americans Work.

[35] (2021). ILO Monitor: COVID-19 and the World of Work. Seventh Edition, Updated Estimates and Analysis. https://www.ilo.org/wcmsp5/groups/public/---dgreports/---dcomm/documents/briefingnote/wcms_767028.pdf.

[36] Rainey R., McCaskill N. (2020). No words for this: 10 million workers file jobless claims in just two weeks. Politico.

[37] Clarke S.A., Phylactou M., Patel B., Mills E.G., Muzi B., Izzi-Engbeaya C., Choudhury S., Khoo B., Meeran K., Comninos A.N. (2021). Normal Adrenal and Thyroid Function in Patients Who Survive COVID-19 Infection. J. Clin. Endocrinol. Metab..

[38] Lui D.T.W., Lee C.H., Chow W.S., Lee A.C.H., Tam A.R., Fong C.H.Y., Law C.Y., Leung E.K.H., To K.K.W., Tan K.C.B. (2021). Thyroid Dysfunction in Relation to Immune Profile, Disease Status, and Outcome in 191 Patients with COVID-19. J. Clin. Endocrinol. Metab..

[39] Rotondi M., Coperchini F., Ricci G., Denegri M., Croce L., Ngnitejeu S.T., Villani L., Magri F., Latrofa F., Chiovato L. (2021). Detection of SARS-COV-2 receptor ACE-2 mRNA in thyroid cells: A clue for COVID-19-related subacute thyroiditis. J. Endocrinol. Investig..

[40] Chen W., Tian Y., Li Z., Zhu J., Wei T., Lei J. (2021). Potential Interaction Between SARS-CoV-2 and Thyroid: A Review. Endocrinology.

[41] Farias S.M., Teixeira O.L., Moreira W., Oliveira M.A., Pereira M.O. (2011). Caracterização dos sintomas físicos de estresse na equipe de pronto atendimento [Characterization of the physical symptoms of stress in the emergency health care team]. Rev. Esc. Enferm. USP.

[42] Posel D., Oyenubi A., Kollamparambil U. (2021). Job loss and mental health during the COVID-19 lockdown: Evidence from South Africa. PLoS ONE.

[43] National Health Care Survey Utilization of Ambulatory Medical Care by Women: United States, 1997–1998, Vital and Health Statistics, 2001, Series 13, Number 149. https://www.researchgate.net/publication/11865009_Utilization_of_Ambulatory_Medical_Care_by_Women_United_States_1997-98.

[44] Castello R., Marco C. (2019). Thyroid diseases and gender. Ital. J. Gend. Specif. Med..

[45] Özdin S., Bayrak Özdin Ş. (2020). Levels and predictors of anxiety, depression and health anxiety during COVID-19 pandemic in Turkish society: The importance of gender. Int. J. Soc. Psychiatry.

[46] Proto E., Quintana-Domeque C. (2021). COVID-19 and mental health deterioration by ethnicity and gender in the UK. PLoS ONE.

[47] Tušl M., Brauchli R., Kerksieck P., Bauer G.F. (2021). Impact of the COVID-19 crisis on work and private life, mental well-being and self-rated health in German and Swiss employees: A cross-sectional online survey. BMC Public Health.

[48] Zürcher S.J., Kerksieck P., Adamus C., Burr C.M., Lehmann A.I., Huber F.K., Richter D. (2020). Prevalence of Mental Health Problems During Virus Epidemics in the General Public, Health Care Workers and Survivors: A Rapid Review of the Evidence. Front. Public Health.

[49] Lai J., Ma S., Wang Y., Cai Z., Hu J., Wei N., Wu J., Du H., Chen T., Li R. (2020). Factors Associated with Mental Health Outcomes Among Health Care Workers Exposed to Coronavirus Disease 2019. JAMA Netw. Open.

[50] Jung H., Jung S.Y., Lee M.H., Kim M.S. (2020). Assessing the Presence of Post-Traumatic Stress and Turnover Intention among Nurses Post-Middle East Respiratory Syndrome Outbreak: The Importance of Supervisor Support. Workplace Health Saf..

[51] Mirzaei A., Molaei B., Habibi-Soola A. (2022). Post-Traumatic Stress Disorder and Its Related Factors in Nurses Caring for COVID-19 Patients. Iran. J. Nurs. Midwifery Res..

[52] Riedel B., Horen S.R., Reynolds A., Hamidian Jahromi A. (2021). Mental Health Disorders in Nurses During the COVID-19 Pandemic: Implications and Coping Strategies. Front. Public Health.

[53] Antonescu I.A., Antonescu A., Miere F., Fritea L., Teușdea A.C., Vicaș L., Vicaș S.I., Brihan I., Domuța M., Zdrinca M. (2021). Evaluation of Wound Healing Potential of Novel Hydrogel Based on Ocimum basilicum and Trifolium pratense Extracts. Processes.

[54] Brihan I., Hălmăjan A., Boda D., Ianoși S.L., Fekete G.L., Zdrincă M. (2020). Role of osteodensitometry in osteoporosis and osteopenia in psoriatic arthritis. Exp. Ther. Med..

[55] Badau A., Rachita A., Sasu C.R., Clipa A. (2018). Motivations and the Level of Practicing Physical Activities by Physio-Kinetotherapy Students. Educ. Sci..

[56] Halmaciu I., Suciu B.A., Trambitas C., Vunvulea V., Ivanescu A., Clipa A., Adascalitei P., Brinzaniuc K., Fodor D. (2018). It is Useful to Use Plastic Anatomical Models in Teaching Human Anatomy ?. Mater. Plast..

[57] Suciu B.A., Halmaciu I., Vunvulea V., Brinzaniuc K. (2018). Is there any correlation between the occurrence of spontaneous pneumothorax and changes in the weather conditions worldwide?. Eur. J. Cardio-Thorac. Surg..

[58] Muhlfay G., Fabian Z., Neagoe R.M., Horvath K.U. (2018). Applications of 3D Planning, Plastic Materials and Additive Manufacturing in Functional Rehabilitations in the Head and Neck Surgery. Mater. Plast..

[59] Neagoe R.M., Sala D., Voidazan S., Bancu S., Kiss L., Suciu H. (2013). Transthoracic versus Transhiatal esophagectomy: A permanent dilemma. our 15-year experience. Chirurgia.

[60] Neagoe R.M., Sala D.T., Roman V., Voidazan S., Pascanu I. (2013). Subtotal parathyroidectomy in the treatment of renal hyperparathyroidism-single center initial experience. Acta Endocrinol..

